# Phenome-wide association studies between *SERINC2* and neuropsychiatric disorders

**DOI:** 10.3389/fpsyt.2024.1420395

**Published:** 2025-01-20

**Authors:** Ping Liu, Xinqun Luo, Liping Cao, Yong Zhang, Jiawu Ji, Xiaoping Wang, Kesheng Wang, Xinghua Pan, Ruilan Yang, Zewen Tan, Yunlong Tan, Chiang-shan Li, Xiaoyun Guo, Zhiren Wang, Xingguang Luo

**Affiliations:** ^1^ Department of Psychosomatic Medicine, People’s Hospital of Deyang City, Deyang, Sichuan, China; ^2^ Department of Neurosurgery, The First Affiliated Hospital, Fujian Medical University, Fuzhou, China; ^3^ Department of Psychiatry, Affiliated Brain Hospital of Guangzhou Medical University, Guangzhou, Guangdong, China; ^4^ Institute of Mental Health, Tianjin Anding Hospital, Mental Health Center of Tianjin Medical University, Tianjin, China; ^5^ Department of Psychiatry, Fujian Medical University Affiliated Fuzhou Neuropsychiatric Hospital, Fuzhou, Fujian, China; ^6^ Department of Neurology, Shanghai Renji Hospital, Shanghai Jiaotong University School of Medicine, Shanghai, China; ^7^ Department of Biobehavioral Health and Nursing Science, College of Nursing, University of South Carolina, Columbia, SC, United States; ^8^ Precision Regenerative Medicine Research Centre, Medical Science Division, and State Key Laboratory of Quality Research in Chinese Medicine, Macau University of Science and Technology, Macau, Macao SAR, China; ^9^ Beijing Huilongguan Hospital, Peking University Huilongguan School of Clinical Medicine, Beijing, China; ^10^ Department of Psychiatry, Yale University School of Medicine, New Haven, CT, United States; ^11^ Shanghai Mental Health Center, Shanghai Jiao Tong University School of Medicine, Shanghai, China

**Keywords:** *SERINC2*, phenome, alcoholism, schizophrenia, OCD, autism, bipolar disorder, mRNA expression

## Abstract

**Objectives:**

*SERINC2* has been associated with alcoholism, bipolar disorder and autism, but the comparability and specificity issues of the findings remain unaddressed. The present study aimed to comprehensively analyze various neuropsychiatric disorders pinpoint the most reliable conditions predisposed by *SERINC2*.

**Methods:**

A total of 2,187 imputed SNPs across *SERINC2* were examined in 1,167,439 subjects from 72 independent cohorts with 18 different neuropsychiatric disorders. SNP-disease associations were tested and then meta-analyzed, followed by FDR correction, to identify significant disease-risk SNPs. Finally, functional studies on the differential *SERINC2* mRNA expression in brains and the potential regulatory effects of disease-risk alleles on *SERINC2* mRNA expression, gray matter volumes (GMVs) of subcortical structures, cortical surface area (SA) and average thickness (TH) were conducted.

**Results:**

In European descent, alcoholism was most significantly associated with *SERINC2* variants (245 SNPs with 5.5×10^-8^≤p ≤ 0.049 and 4.9×10^-5^≤q ≤ 0.034) that were largely shared across cocaine dependence, marijuana dependence, nicotine dependence, polysubstance dependence, schizophrenia, OCD, and autism (8.2×10^-8^≤p ≤ 0.050 and 1.9×10^-5^≤q ≤ 0.049); in Chinese population, bipolar disorder was also significantly associated with *SERINC2* variants (10 SNPs: 1.3×10^-4^≤p ≤ 4.7×10^-4^ and 0.025≤q ≤ 0.031). Furthermore, the disease-risk alleles had highly similar regulatory effects on mRNA expression (8.1×10^-7^≤p ≤ 0.046), subcortical GMVs (7.0×10^-4^≤p ≤ 0.048) and cortical TH and SA (1.3×10^-3^≤p ≤ 0.050) in brains across alcoholism, schizophrenia, OCD and autism. The bipolar disorder-risk alleles had these regulatory effects but with different effect patterns. Finally, *SERINC2* mRNA was differentially expressed in several brain regions between alcoholism or schizophrenia and controls.

**Conclusion:**

*SERINC2* is primarily linked to substance use disorders, schizophrenia, OCD, autism and bipolar disorder, not only statistically but also biologically.

## 1 Introduction

Several genome-wide association studies (GWAS) have identified serine incorporator 2 gene *(SERINC2)* as a genome-wide significant risk gene for alcohol dependence in European descent (1–3). Further, the common *SERINC2* variants and rare *SERINC2* variant constellations have both been reported to be “specific” to risk for alcohol dependence in European descent among 12 diverse neuropsychiatric disorders (1, 4). Following these studies, *SERINC2* variants have also been associated to bipolar disorder (BP) in a Chinese population (5) and autism spectrum disorder (ASD) in a Thai population (6). In a family-based sample with multiple BP-affected Chinese pedigrees, whole-exome sequencing identified several rare *SERINC2* variants significantly associated with BP, which was confirmed by a larger population-based Chinese cohort (5). In a Thai sample with ASD, microarray experiment identified a rare *de novo* duplication of a pathogenic copy number variation (CNV) in *SERINC2* predisposing risk for ASD (6).

*SERINC2* encodes a transmembrane protein that facilitates incorporation of serine into phosphatidylserine and sphingolipids (7). The concentration of sphingolipids is highest in the brain; they play important roles in neural plasticity, signaling and axonal guidance (8). MRI image results show that *SERINC2* variants affect the brain structures such as white matter volume of cerebellum (5). These physiological functions support a potential role of *SERINC2* in multiple neuropsychiatric, neurodegenerative and neurodevelopmental diseases such as alcoholism, bipolar disorder and autism.

However, whether *SERINC2* is most significantly associated with alcoholism, whether *SERINC2* is also associated with alcoholism-comorbid disorders, whether *SERINC2* is associated with more other neuropsychiatric disorders than alcoholism, BP and ASD, and how to make the findings from different studies with diverse study design, genetic marker sets, and analytic methodologies comparable remains to be answered. To answer these questions, here, we proposed a single study to comprehensively analyze a huge dataset harboring a total of 1,167,439 subjects from 72 independent cohorts with 18 different neuropsychiatric disorders, by standardizing study design, genetic marker sets, and analytic methodologies across phenome.

## 2 Materials and methods

### 2.1 Subjects

We conducted a comprehensive analysis involving 1,167,439 participants from 72 independent cohorts, each representing one of 18 distinct neuropsychiatric disorders. These disorders spanned a broad spectrum, including schizophrenia (12 cohorts), bipolar disorder (BP; 10 cohorts), major depression (7 cohorts), autism (1 cohort), alcoholism (4 cohorts), nicotine dependence (7 cohorts), cocaine dependence (2 cohorts), marijuana dependence (2 cohorts), opioid dependence (3 cohorts), ADHD (7 cohorts), Alzheimer’s disease (2 cohorts), Parkinson’s disease (4 cohorts), multiple sclerosis (4 cohorts), amyotrophic lateral sclerosis (1 cohort), and stroke (3 cohorts). In particular, in European populations, there were nine separate cohorts dedicated to the study of substance dependence, including two cohorts for alcoholism, one cohort for cocaine dependence, one cohort for marijuana dependence, four cohorts for nicotine dependence, and one cohort for multi-substance dependence; and there were eight separate cohorts for schizophrenia, one for OCD, and one for autism. In Chinese populations, there were two separate cohorts for bipolar disorder. All participants provided written informed consent or assent, and all study procedures were rigorously reviewed and approved by the Human Investigation Committee of the respective institutions.

**Supplementary Table S1** provides comprehensive information for each cohort, including sample types, microarray platforms, cohort numbers, dataset names, diagnoses, ethnicities, study designs, sample sizes, grant support numbers, principal investigators, references, and dbGaP accession numbers. Detailed demographic data for these cohorts have been previously published and can be accessed via the PMID# listed in **Supplementary Table S1**.

### 2.2 Genotyping and imputation

All study participants underwent genotyping using microarray technologies; however, different cohorts were genotyped using distinct array panels. To ensure consistency in the genetic marker sets across all cohorts, we performed imputation for untyped SNPs across the entire *SERINC2* (5’, ORF, and 3’) separately for each ethnicity, utilizing reference panels from the 1000 Genomes Project and HapMap3 Project. The imputation was conducted using the IMPUTE2 program (9), following a well-established protocol from previous literature (10). This rigorous approach ensured the accuracy and quality of the imputed genotype data. For internal cross-validation, each cohort was divided into case and control groups, and imputation was performed separately for cases, controls, and the total group. Only imputed SNPs with high imputation accuracy (INFO > 0.8) across all three groups were included in the following SNP-disease association analysis. After the association analysis, the phase of each imputed risk SNP was re-checked across the groups to further confirm the accuracy of imputation.

### 2.3 Summary of analytic strategy

Before conducting the association analysis, we thoroughly cleaned the phenotype and genotype data, as previously described (10, 11). The SNP-disease associations within each cohort were analyzed using the PLINK software (12), incorporating appropriate analytical approaches. To account for population stratification and admixture (11), the first 10 principal components (PCs) of ancestry were included as covariates. The p-values from these associations were then combined through meta-analysis to generate combined p-values for each of the 18 disorders across three distinct ethnic groups: Chinese, Europeans, and African Americans. To further ensure the robustness of our findings, we calculated q-values, adjusting the combined p-values using an optimized false discovery rate (FDR) approach (13) to identify significant disease-risk alleles.

We also examined *SERINC2* mRNA expression in postmortem human brains using the GTEx dataset (14) and performed a cis-eQTL analysis to explore the regulatory effects of disease-risk variants on SERINC2 expression. To support the potential functional significance of the *SERINC2* risk SNPs, we conducted differential expression analysis of *SERINC2* mRNA across 10 independent cohorts of postmortem brain tissues. These cohorts included one for alcoholism (15), one for cocaine dependence (16), one for nicotine dependence (17), two for bipolar disorder (18, 19), and five for schizophrenia (19–22), along with respective controls. Detailed information on these cohorts is available in the published literature (15–22).

Finally, we assessed the regulatory effects of disease-risk alleles on intracranial volume (ICV), subcortical grey matter volumes (GMVs), cortical surface area (SA), and cortical thickness (TH) to explore their potential biological functions. Comprehensive details on the data cleaning procedures, SNP-disease association analysis, differential expression of *SERINC2* mRNA, *cis*-eQTL analysis, and the analysis of regulatory effects on ICV, GMVs, cortical SA, and average TH can be found in the Supplementary Materials and Methods of the study by Guo et al. (2024) (23).

## 3 Results

### 3.1 SNP-disease association

#### 3.1.1 SNP-disease association in each cohort

A total of 2,187 imputed SNPs across 5’, open reading frame and 3’ of *SERINC2* were examined in all 72 cohorts. Variants numbered from 1 to 313 were found to be nominally associated with a disease in each of the 72 cohorts (8.0×10^-11^≤p<0.05), except for cohorts #53, #57, and #58 (ADHD) and #71 (Stroke), where no significant associations were observed (**Supplementary Table S1**).

#### 3.1.2 SNP-disease association for each disease

After meta-analysis of 2,187 SNP-disease associations for each of all 18 neuropsychiatric disorders within the same ethnicity, variants ranging from 2 to 251 remained nominally associated with their respective diseases (meta: 5.9×10^-9^≤p ≤ 0.028; some data are provided in **Table 1**), except for Stroke in Europeans (p>0.05; data not shown).

**Table 1A:** Significant associations between *SERINC2* variants and neuropsychiatric disorders in European descent.

SNP	Position	Risk	Protective	Z-score	Alcoholism		Cocaine Dep.		Marijuana Dep.		Nicotine Dep.		Schizophrenia		OCD	
					p-value	q-value	p-value	q-value	p-value	q-value	p-value	q-value	p-value	q-value	p-value	q-value
	(B37)	Allele	Allele	(meta)	(meta)	(FDR)	(meta)	(FDR)	(meta)	(FDR)	(meta)	(FDR)	(meta)	(FDR)	(meta)	(FDR)
Top 10 associations for Alcoholism in Europeans																
rs12132936	31895931	g	a	5.435	**5.5×10^-8^**	**4.9×10^-6^**	3.5×10^-4^	1.6×10^-3^	2.3×10^-4^	1.7×10^-3^	>0.05	>0.05	5.8×10^-5^	3.4×10^-4^	0.023	0.024
rs4949403	31898279	a	c	5.422	**5.9×10^-8^**	**4.9×10^-6^**	1.1×10^-4^	6.6×10^-4^	1.6×10^-4^	1.7×10^-3^	>0.05	>0.05	3.6×10^-5^	2.2×10^-4^	0.012	0.024
rs4949401	31898162	c	t	5.054	**4.3×10^-7^**	**1.7×10^-5^**	4.0×10^-4^	1.7×10^-3^	7.2×10^-4^	1.7×10^-3^	>0.05	>0.05	3.0×10^-5^	2.0×10^-4^	0.014	0.024
rs10914383	31894402	g	a	5.038	**4.7×10^-7^**	**1.7×10^-5^**	2.1×10^-4^	1.2×10^-3^	1.2×10^-3^	1.7×10^-3^	>0.05	>0.05	1.7×10^-5^	1.4×10^-4^	0.015	0.024
rs4949209	31897963	c	t	5.028	**5.0×10^-7^**	**1.7×10^-5^**	3.5×10^-4^	1.6×10^-3^	7.3×10^-4^	1.7×10^-3^	>0.05	>0.05	2.2×10^-5^	1.6×10^-4^	0.016	0.024
rs1320584	31897063	g	t	4.984	**6.2×10^-7^**	**1.7×10^-5^**	3.0×10^-4^	1.5×10^-3^	9.7×10^-4^	1.7×10^-3^	>0.05	>0.05	1.4×10^-5^	1.2×10^-4^	0.014	0.024
rs12037108	31895394	c	t	4.944	**7.6×10^-7^**	**1.8×10^-5^**	3.5×10^-4^	1.6×10^-3^	1.8×10^-3^	2.0×10^-3^	>0.05	>0.05	2.5×10^-5^	1.8×10^-4^	0.010	0.024
rs10798850	31892148	a	t	4.826	**1.4×10^-6^**	**2.6×10^-5^**	8.5×10^-5^	6.6×10^-4^	1.0×10^-3^	1.7×10^-3^	>0.05	>0.05	2.6×10^-5^	1.8×10^-4^	0.017	0.024
rs4478858	31883925	t	c	4.823	**1.4×10^-6^**	**2.6×10^-5^**	3.1×10^-4^	1.5×10^-3^	6.4×10^-4^	1.7×10^-3^	5.0×10^-3^	0.029	2.6×10^-5^	1.8×10^-4^	0.042	0.026
rs6690908	31910089	t	c	4.556	**5.2×10^-6^**	**8.8×10^-5^**	>0.05	>0.05	0.012	3.5×10^-3^	9.0×10^-4^	0.026	>0.05	>0.05	0.031	0.024
Top 10 associations for Schizophrenia in Europeans																
rs114737875	31859959	a	t	5.362	>0.05	>0.05	>0.05	>0.05	>0.05	>0.05	>0.05	>0.05	**8.2×10^-8^**	**1.9×10^-5^**	>0.05	>0.05
rs12117387	31862346	a	g	5.326	>0.05	>0.05	>0.05	>0.05	>0.05	>0.05	>0.05	>0.05	**1.0×10^-7^**	**1.9×10^-5^**	>0.05	>0.05
rs7515829	31856064	g	a	5.282	>0.05	>0.05	>0.05	>0.05	>0.05	>0.05	>0.05	>0.05	**1.3×10^-7^**	**1.9×10^-5^**	>0.05	>0.05
rs6425745	31864323	g	a	5.266	>0.05	>0.05	>0.05	>0.05	>0.05	>0.05	>0.05	>0.05	**1.4×10^-7^**	**1.9×10^-5^**	>0.05	>0.05
rs10914374	31872581	g	t	5.023	0.022	0.020	9.3×10^-5^	6.6×10^-4^	5.6×10^-3^	3.1×10^-3^	>0.05	>0.05	**5.1×10^-7^**	**3.7×10^-5^**	>0.05	>0.05
rs4949402	31898234	t	c	5.013	6.7×10^-3^	0.011	2.6×10^-4^	1.4×10^-3^	5.5×10^-4^	1.7×10^-3^	>0.05	>0.05	**5.4×10^-7^**	**3.7×10^-5^**	>0.05	>0.05
rs10798848	31874162	a	g	4.956	0.017	0.018	1.2×10^-4^	6.9×10^-4^	0.010	3.5×10^-3^	6.5×10^-3^	0.029	**7.2×10^-7^**	**3.7×10^-5^**	0.040	0.026
rs4949393	31851875	g	a	4.954	1.4×10^-4^	5.0×10^-4^	3.0×10^-3^	8.9×10^-3^	3.7×10^-3^	3.1×10^-3^	7.9×10^-3^	0.032	**7.3×10^-7^**	**3.7×10^-5^**	>0.05	>0.05
rs12145450	31862949	c	t	4.941	0.024	0.021	7.4×10^-5^	6.6×10^-4^	5.5×10^-3^	3.1×10^-3^	>0.05	>0.05	**7.8×10^-7^**	**3.7×10^-5^**	0.029	0.024
rs12563669	31858067	a	g	4.937	3.0×10^-4^	8.6×10^-4^	3.5×10^-3^	0.010	4.5×10^-3^	3.1×10^-3^	1.8×10^-3^	0.026	**8.0×10^-7^**	**3.7×10^-5^**	0.027	0.024
Top 10 associations for OCD in Europeans																
rs7545902	31884936	c	g	1.095*	>0.05	>0.05	>0.05	>0.05	>0.05	>0.05	>0.05	>0.05	2.6×10^-5^	1.8×10^-4^	**1.3×10^-6^**	**6.5×10^-5^**
rs4949397	31879462	c	g	1.101*	5.4×10^-4^	1.5×10^-3^	4.4×10^-3^	0.012	1.6×10^-3^	1.9×10^-3^	>0.05	>0.05	1.5×10^-5^	1.2×10^-4^	**2.3×10^-6^**	**6.5×10^-5^**
rs4949396	31879417	t	c	1.101*	3.3×10^-4^	9.5×10^-4^	4.4×10^-3^	0.012	1.6×10^-3^	1.9×10^-3^	>0.05	>0.05	1.7×10^-5^	1.4×10^-4^	**2.3×10^-6^**	**6.5×10^-5^**
rs1977657	32031651	a	t	1.205*	>0.05	>0.05	>0.05	>0.05	>0.05	>0.05	>0.05	>0.05	0.017	0.049	**3.9×10^-6^**	**8.2×10^-5^**
rs4949395	31879284	c	t	1.131*	0.018	0.019	>0.05	>0.05	0.049	8.4×10^-3^	>0.05	>0.05	3.2×10^-5^	2.1×10^-4^	**5.8×10^-6^**	**9.9×10^-5^**
rs10798849	31876615	t	c	1.094*	1.7×10^-5^	1.8×10^-4^	3.9×10^-3^	0.011	1.6×10^-3^	1.9×10^-3^	>0.05	>0.05	2.2×10^-5^	1.6×10^-4^	**7.6×10^-6^**	**1.1×10^-4^**
rs56332792	31974308	t	g	1.160*	7.2×10^-3^	0.012	>0.05	>0.05	>0.05	>0.05	>0.05	>0.05	>0.05	>0.05	**2.9×10^-4^**	**3.6×10^-3^**
rs10753251	31975820	g	a	1.097*	0.019	0.019	>0.05	>0.05	9.5×10^-3^	3.5×10^-3^	>0.05	>0.05	>0.05	>0.05	**9.3×10^-4^**	**9.8×10^-3^**
rs10798861	31975832	g	a	1.095*	0.020	0.019	>0.05	>0.05	9.5×10^-3^	3.5×10^-3^	>0.05	>0.05	>0.05	>0.05	**1.1×10^-3^**	**0.011**
rs2839939	31881637	c	t	1.065*	0.011	0.015	5.3×10^-4^	2.2×10^-3^	9.0×10^-4^	1.7×10^-3^	>0.05	>0.05	1.1×10^-4^	6.3×10^-4^	**1.2×10^-3^**	**0.011**
Top 2 associations for Autism in Europeans																
rs10798856	31951092	g	a	1.193*	>0.05	>0.05	>0.05	>0.05	>0.05	>0.05	3.5×10^-3^	0.026	>0.05	>0.05	**7.2×10^-5^**	**5.3×10^-3^**
rs10158864	31977299	g	a	1.238*	>0.05	>0.05	>0.05	>0.05	0.012	3.5×10^-3^	>0.05	>0.05	>0.05	>0.05	**2.0×10^-7^**	**2.9×10^-5^**

Bold values, only top 10 associations are listed for each disease; Meta, meta-analysis; q value, adjusted p values by false discovery rate (FDR). *, odd ratio (OR) values.

Followed by FDR correction, alcoholism was most significantly associated with *SERINC2* variants in EAs (245 SNPs with 5.5×10^-8^≤p ≤ 0.049 and 4.9×10^-5^≤q ≤ 0.034; **Table 1A**). Interestingly, multiple other substance dependence in EAs was significantly associated with *SERINC2* variants too, including cocaine dependence (107 SNPs with 2.6×10^-5^≤p ≤ 0.020 and 6.6×10^-4^≤q ≤ 0.046; **Table 1A**), marijuana dependence (213 SNPs with 1.6×10^-4^≤p ≤ 0.049 and 1.7×10^-3^≤q ≤ 8.5×10^-3^; **Table 1A**), nicotine dependence (85 SNPs with 6.9×10^-4^≤p ≤ 0.016 and 0.026≤q ≤ 0.049; **Table 1A**), and multi-substance dependence (rs28742121 and rs28759069: p=1.7×10^-3^ and q=0.033; data not shown).

The second most significant disease associated with *SERINC2* variants was schizophrenia in EAs (187 SNPs with 8.2×10^-8^≤p ≤ 0.018 and 1.9×10^-5^≤q ≤ 0.049; **Table 1A**), followed by OCD in EAs (150 SNPs with 1.3×10^-6^≤p ≤ 0.050 and 6.5×10^-5^≤q ≤ 0.028; **Table 1A**). Additionally, a much smaller number of *SERINC2* variants was significantly associated with autism in EAs (rs10798856 and rs10158864: 2.0×10^-7^≤p ≤ 7.2×10^-5^ and 2.9×10^-5^≤q ≤ 5.3×10^-3^; **Table 1A**) and bipolar disorder in Chinese population (10 SNPs with 1.3×10^-4^≤p ≤ 4.7×10^-4^ and 0.025≤q ≤ 0.031; **Table 1B**). Notably, these risk variants were largely shared across various substance dependence, schizophrenia, OCD and autism (**Table 1A**), but not bipolar disorder (**Table 1B**).

**Table 1B:** Significant associations between *SERINC2* variants and bipolar disorder in Asian descent.

SNP	Position	Risk	Protective	Z-score	p-value	q-value
	(B37)	Allele	Allele	(meta)	(meta)	(FDR)
rs12734726	31975609	c	t	3.717	2.0×10^-4^	0.025
rs56214663	32006715	g	a	3.517	4.4×10^-4^	0.031
rs60788028	32009880	g	a	3.670	2.4×10^-4^	0.025
rs58654289	32004752	t	g	3.650	2.6×10^-4^	0.025
rs72881860	31989332	c	t	3.496	4.7×10^-4^	0.031
rs4949429	32003454	c	g	3.693	2.2×10^-4^	0.025
rs4949430	32003488	c	t	3.693	2.2×10^-4^	0.025
rs59170272	32005323	t	c	3.830	1.3×10^-4^	0.025
rs72881892	32005636	t	a	3.693	2.2×10^-4^	0.025
rs10798871	32009436	a	g	3.578	3.5×10^-4^	0.029

Meta, meta-analysis; q value, adjusted p values by false discovery rate (FDR).

Finally, the phase of each imputed risk SNP listed in **Table 1** is the same across cases, controls, and total group, confirming the accuracy of imputation.

### 3.2 Differential expression of SERINC2 mRNA in brains

**Figure 1:**
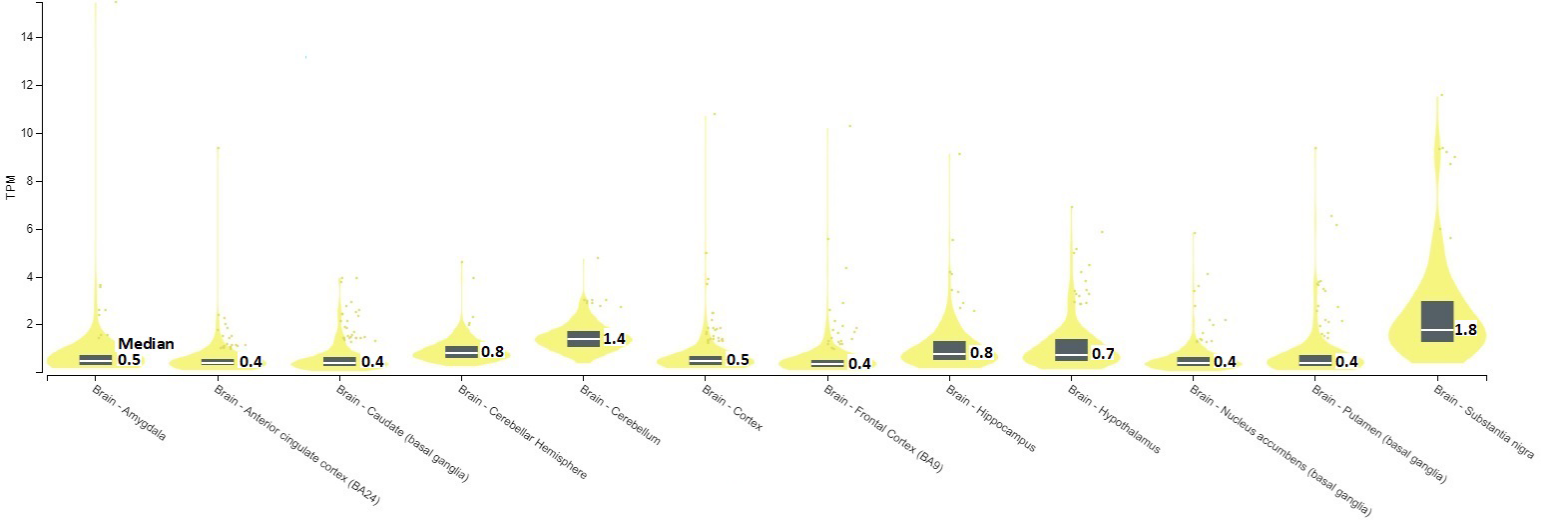
*SERINC2* mRNA expression in brains [TPM, Transcripts Per Million; Median, median TPM value; TPM>1 indicates the presence of mRNA expression].

In GTEx cohort, SERINC2 mRNA is significantly expressed in two brain regions, including substantia nigra (median TPM = 1.8) and cerebellum (1.4) (**Figure 1**).

Three independent cohorts showed *SERINC2* mRNA was differentially expressed in several other brains between alcoholism or schizophrenia and controls. The expression in hippocampus was increased in alcoholism (p=0.010), but the expression in neurons was decreased in schizophrenia in two cohorts (p=0.041 and 0.039, respectively), when compared to controls (**Table 2**). No differential expression was detected in other 7 cohorts (data not shown).

**Table 2:** Differential expression of *SERINC2* mRNA in brains with alcoholism or schizophrenia.

	Cohort 1	Cohort 2	Cohort 3
Organism	Human	Human	Human
Brain region	Hippocampus	Neuron	Neuron
Dataset names	GEO	GEO	GEO
Accession number	GSE44456	GSE12679	GSE25673
References	PMID: 23981442	PMID: 19088852	PMID: 21490598
Experiment methods	Affymetrix Human	Affymetrix Human	Affymetrix Human
	Genome U133A Array	Genome U133A Array	Genome U133A Array
Measurement of expression	Log2(normalized intensity)	Log2(normalized intensity)	Log2(normalized intensity)
Control subjects:			
Phenotype	healthy	healthy	healthy
Tissue types	post-mortem brain tissue	post-mortem brain tissue	post-mortem brain tissue
Sample sizes	19	6	12
Expression levels	6.2 ± 0.18	1.1 ± 0.22	7.5 ± 0.14
Case subjects:			
Phenotype	alcoholism	schizophrenia	schizophrenia
Tissue types	post-mortem brain tissue	post-mortem brain tissue	post-mortem brain tissue
Sample sizes	20	5	12
Expression levels	6.4 ± 0.18	0.8 ± 0.21	7.2 ± 0.32
p-values for t-test	0.010	0.041	0.039

GEO, Gene Expression Omnibus database.

### 3.3 SNP-mRNA associations: cis-eQTL analysis

The disease-risk alleles had highly similar association patterns with mRNA expression in brain regions and effect directions across alcoholism, schizophrenia, OCD and autism (**Table 3A**). In substantia nigra, the disease-risk alleles decreased *SERINC2* mRNA expression (5.3×10^-3^≤p ≤ 0.046), but in other brain regions, including anterior cingulate cortex, cerebellar hemisphere, cortex and hippocampus, they increased mRNA expression (8.1×10^-7^≤p ≤ 0.042; **Table 3A**).

**Table 3A:** Associations between disease-risk alleles and *SERINC2* mRNA expression in brains.

Associated disorders	SNP	Disease-risk alleles	Decreasing mRNA			Increasing mRNA by disease-risk alleles								
			Effective alleles	Substantia nigra		Effective allele	Anterior cingulate		Cerebellar hemisphere		Cortex		Hippocampus	
				NES	P		NES	P	NES	P	NES	P	NES	P
Alcoholism	rs12132936	g	a	0.190	0.046	g	0.250	3.6×10^-3^			0.330	8.1×10^-7^	0.170	0.022
	rs4949403	a	c			a	0.260	2.4×10^-3^			0.340	1.1×10^-6^	0.210	2.9×10^-3^
	rs4949401	c	t			c	0.260	2.1×10^-3^			0.340	1.1×10^-6^	0.210	2.9×10^-3^
	rs10914383	g	a			g	0.250	3.3×10^-3^			0.340	1.6×10^-6^	0.160	0.026
	rs4949209	c	t			c	0.270	2.1×10^-3^			0.340	7.1×10^-7^	0.220	2.9×10^-3^
	rs1320584	g	t			g	0.260	2.5×10^-3^	0.160	0.042	0.320	2.2×10^-6^	0.210	2.9×10^-3^
	rs12037108	c	t	0.190	0.046	c	0.250	3.6×10^-3^			0.330	8.1×10^-7^	0.170	0.022
	rs10798850	a	t	0.190	0.046	a	0.250	3.5×10^-3^			0.330	8.1×10^-7^	0.170	0.022
	rs4478858	t	c			t	0.250	2.3×10^-3^			0.300	6.5×10^-6^	0.180	0.014
	rs6690908	t	c			t					0.170	0.021	0.250	2.0×10^-3^
Schizophrenia	rs12117387	a	g	0.320	0.010	a					0.210	6.7×10^-3^		
	rs7515829	g	a	0.350	5.3×10^-3^	g					0.210	6.7×10^-3^		
	rs10914374	g	t	0.320	0.010	g					0.210	6.7×10^-3^		
	rs4949402	t	c			t	0.270	2.1×10^-3^			0.340	7.8×10^-7^	0.220	2.9×10^-3^
	rs10798848	a	g	0.320	0.010	a					0.200	0.010		
	rs4949393	g	a	0.230	0.019	g			0.160	0.043	0.270	9.3×10^-5^		
	rs12145450	c	t	0.320	0.010	c					0.210	6.7×10^-3^		
	rs12563669	a	g	0.230	0.021	a	0.180	0.042			0.280	6.7×10^-5^		
OCD	rs7545902	c	g			c	0.260	1.9×10^-3^	0.160	0.041	0.310	7.7×10^-6^	0.160	0.026
	rs4949397	c	g	0.210	0.026	c	0.210	0.013	0.170	0.027	0.300	1.1×10^-5^	0.160	0.025
	rs4949396	t	c	0.210	0.026	t	0.210	0.013	0.170	0.026	0.290	2.7×10^-5^	0.160	0.023
	rs1977657	a	t			a			0.220	0.013				
	rs4949395	c	t	0.210	0.026	c	0.210	0.013	0.170	0.030	0.290	2.3×10^-5^	0.160	0.023
	rs10798849	t	c	0.210	0.026	t	0.210	0.013	0.170	0.030	0.290	2.3×10^-5^	0.160	0.023
	rs56332792	t	g			t			0.160	0.038				
	rs10753251	g	a			g			0.200	8.6×10^-3^				
	rs10798861	g	a			g			0.200	8.6×10^-3^				
	rs2839939	c	t			c	0.260	2.2×10^-3^			0.310	4.4×10^-6^	0.180	0.016
Autism	rs10158864	g	a			g			0.200	8.6×10^-3^				

NES, normalized effect size.

The bipolar disorder-risk alleles decreased *SERINC2* mRNA expression in caudate, cerebellar hemisphere and hypothalamus (0.007≤p ≤ 0.044) but increased it in frontal cortex (0.024≤p ≤ 0.027; **Table 3B**). Additionally, one autism-risk allele decreased *SERINC2* mRNA expression in cerebellar hemisphere and cortex (1.6×10^-4^≤p ≤ 0.020; **Table 3B**).

**Table 3B:** Associations between disease-risk alleles and *SERINC2* mRNA expression in brains.

Associated disorders	SNP	Disease-risk alleles	Decreasing mRNA by disease-risk alleles									Increasing mRNA		
			Effective alleles	Caudate		Cerebellar hemisphere		Cortex		Hypothalamus		Effective allele	Frontal Cortex	
				NES	P	NES	P	NES	P	NES	P		NES	P
Autism	rs10798856	g	a			0.200	0.020	0.260	1.6×10^-4^			g		
Bipolar	rs12734726	c	t							0.300	0.028	c		
	rs56214663	g	a	0.560	0.043	0.500	0.044					g	0.530	0.024
	rs60788028	g	a	0.720	0.014							g	0.530	0.027
	rs58654289	t	g	0.440	0.029	0.530	0.007					t		
	rs72881860	c	t	0.560	0.043	0.500	0.044					c	0.530	0.024
	rs4949429	c	g	0.440	0.029	0.530	0.007					c		
	rs4949430	c	t	0.440	0.029	0.530	0.007					c		
	rs59170272	t	c	0.440	0.029	0.530	0.007					t		
	rs72881892	t	a	0.440	0.029	0.530	0.007					t		
	rs10798871	a	g	0.440	0.029	0.530	0.007					a		

NES, normalized effect size.

### 3.4 The disease-risk alleles decreased the ICV and the GMV of caudate and pallidum but increased the GMVs of accumbens and putamen

The disease-risk alleles had highly similar association patterns with GMVs of caudate and putamen across alcoholism, schizophrenia, OCD and autism (**Table 4A**). These alleles decreased caudate GMVs across two independent cohorts (7.0×10^-4^≤p ≤ 0.048) and increased putamen GMV in one cohort (9.1×10^-3^≤p ≤ 0.041; **Table 4A**). Furthermore, the alcoholism-risk allele T of rs6690908 decreased ICV in one cohort (p=0.017) but the schizophrenia-risk alleles increased accumbens GMVs across two independent cohorts (0.011≤p ≤ 0.017; **Table 4A**). Additionally, the bipolar-risk alleles decreased caudate and pallidum GMVs (0.026≤p ≤ 0.048; **Table 4B**).

**Table 4A:** p-values for SNP-GMV associations in subcortical structures.

Associated disorders	SNP	Disease-risk allele*	Decreasing GMVs by disease-risk alleles				Increasing GMVs by disease-risk alleles			
			Effective Allele**	CHARGE	“unrestricted”	ENIGMA2	Effective Allele	“restricted”	ENIGMA2	“restricted”
				ICV	Caudate	Caudate		Accumbens	Accumbens	Putamen
Alcoholism	rs12132936	g	a		5.8×10^-3^	0.025	g			0.016
	rs4949403	a	c		7.6×10^-3^	0.030	a			0.013
	rs4949401	c	t		8.4×10^-3^	0.030	c			0.012
	rs10914383	g	a		7.0×10^-3^	0.026	g			0.016
	rs4949209	c	t		7.8×10^-3^	0.028	c			0.015
	rs1320584	g	t		6.6×10^-3^	0.027	g			0.016
	rs12037108	c	t		6.0×10^-3^	0.026	c			0.016
	rs10798850	a	t		6.5×10^-3^		a			0.016
	rs4478858	t	c		8.4×10^-3^	0.028	t			0.012
	rs6690908	t	c	0.017			t			0.041
Schizophrenia	rs114737875	a	t				a	0.014		
	rs12117387	a	g			0.013	a		0.014	0.034
	rs7515829	g	a		0.043	9.2×10^-3^	g		0.016	0.040
	rs6425745	g	a			0.013	g	0.017	0.015	
	rs10914374	g	t			0.016	g		0.013	0.039
	rs4949402	t	c		8.3×10^-3^	0.032	t			0.012
	rs10798848	a	g			0.022	a		0.011	0.039
	rs4949393	g	a		7.0×10^-4^	0.012	g			0.045
	rs12145450	c	t			0.013	c		0.014	0.036
	rs12563669	a	g		8.1×10^-4^	0.016	a			0.040
OCD	rs7545902	c	g		8.6×10^-3^		c			0.013
	rs4949397	c	g		1.1×10^-3^		c			0.027
	rs4949396	t	c		9.7×10^-4^		t			0.028
	rs4949395	c	t		8.2×10^-4^	0.048	c			0.034
	rs10798849	t	c		8.4×10^-4^		t			0.039
	rs10753251	g	a				g			0.028
	rs10798861	g	a				g			0.028
	rs2839939	c	t		9.3×10^-3^	0.022	c			9.1×10^-3^
Autism	rs10158864	g	a				g			0.026

*disease-risk alleles increase risk for diseases (**Table 1**); **effective alleles increase GMVs. CHARGE, CHARGE-ENIGMA cohort; ENIGMA2, ENIGMA2 cohort; GMV, grey matter volume; ICV, intracranial volume.

**Table 4B:** p-values for SNP-GMV associations in subcortical structures.

Associated disorder	SNP	Disease-risk allele*	Decreasing GMVs		
			Effective Allele**	ENIGMA2	ENIGMA2
				Pallidum	Caudate
Bipolar	rs12734726	c	t	>0.05	>0.05
	rs56214663	g	a	0.038	>0.05
	rs60788028	g	a	0.026	0.048
	rs58654289	t	g	0.045	>0.05
	rs72881860	c	t	0.043	>0.05
	rs4949429	c	g	–	–
	rs4949430	c	t	0.046	>0.05
	rs59170272	t	c	0.044	>0.05
	rs72881892	t	a	–	–
	rs10798871	a	g	0.031	>0.05

* disease-risk alleles increase risk for diseases (**Table 1A**); ** effective alleles increase GMVs. ENIGMA2, ENIGMA2 cohort; “-”, missing values.

### 3.5 The disease-risk alleles regulated the cortical SA and TH of multiple brain regions

The disease-risk alleles had highly similar association patterns with cortical SA/TH across alcoholism, schizophrenia, OCD and autism (**Table 5**). These alleles decreased SA/TH of fusiform, inferior temporal, precuneus, superior parietal, superior temporal, supramarginal, transverse temporal, caudal anterior cingulate, entorhinal, parahippocampal, parstriangularis and temporal pole cortices (1.3×10^-3^≤p ≤ 0.048; **Table 5A**) and increased SA/TH of bankssts, caudal middle frontal, insula, lateralorbitofrontal, middle temporal, paracentral, parsopercularis, parsorbitalis, parstriangularis, posterior cingulate, precentral, caudal middle frontal, cuneus, lateral occipital, superior temporal and superior parietal cortices (5.4×10^-3^≤p ≤ 0.050; **Table 5B**).

**Table 5A:** p-values for negative associations between disease-risk alleles and TH/SA in brains.

SNP	Risk allele	Effective allele	SA							TH						
			ENG3	ENG3	ENG3	ENG3	ENG3	ENG3	ENG3	ENG3	UKBB	ENG3	ENG3	ENG3	UKBB	ENG3
			Fusi-form	Inferior-temporal	Pre-cuneus	Superior-parietal	Superior-temporal	Supra-marginal	Transverse-temporal	Caudal-anterior-cingulate	Caudal-anterior-cingulate	Entor-hinal	Para-hippo-campal	Pars-triangularis	Pars-triangularis	Temporal-pole
rs12132936	g	a		0.031		9.4×10^-3^			6.4×10^-3^		0.010	2.9×10^-3^	0.022		5.2×10^-3^	3.9×10^-3^
rs4949403	a	c		0.030		0.010			1.7×10^-3^		0.013	2.7×10^-3^	0.015		3.1×10^-3^	4.8×10^-3^
rs4949401	c	t		0.033		0.010			1.8×10^-3^		0.012	2.6×10^-3^	0.015		3.2×10^-3^	5.4×10^-3^
rs10914383	g	a		0.042		0.010			3.5×10^-3^		0.013	2.6×10^-3^	0.023		4.0×10^-3^	6.0×10^-3^
rs4949209	c	t		0.027		9.0×10^-3^			4.6×10^-3^		0.016	2.3×10^-3^	0.017		3.4×10^-3^	5.3×10^-3^
rs1320584	g	t		0.032		9.2×10^-3^			3.6×10^-3^		0.013	2.3×10^-3^	0.018		3.4×10^-3^	5.2×10^-3^
rs12037108	c	t		0.034		0.010			5.5×10^-3^		0.011	3.2×10^-3^	0.024		4.6×10^-3^	3.7×10^-3^
rs10798850	a	t		0.038		0.010			4.8×10^-3^		0.010	2.8×10^-3^	0.025		5.0×10^-3^	3.4×10^-3^
rs4478858	t	c				0.013		0.046	3.1×10^-3^		9.4×10^-3^	5.1×10^-3^	0.028		3.6×10^-3^	3.1×10^-3^
rs6690908	t	c				0.017		5.2×10^-3^		0.038	0.014					
rs12117387	a	g										4.2×10^-3^		0.023	1.8×10^-3^	
rs7515829	g	a										4.4×10^-3^		0.017	1.4×10^-3^	
rs6425745	g	a										4.2×10^-3^		0.023	1.8×10^-3^	
rs10914374	g	t										4.0×10^-3^		0.023	1.7×10^-3^	
rs4949402	t	c		0.031		8.7×10^-3^			2.7×10^-3^		0.012	2.5×10^-3^	0.014		3.2×10^-3^	5.2×10^-3^
rs10798848	a	g										3.6×10^-3^		0.020	1.3×10^-3^	
rs4949393	g	a					0.040		1.9×10^-3^		8.7×10^-3^	6.7×10^-3^			0.026	0.042
rs12145450	c	t										4.5×10^-3^		0.028	1.8×10^-3^	
rs12563669	a	g					0.044		3.0×10^-3^		0.012	9.3×10^-3^			0.039	
rs7545902	c	g									9.4×10^-3^				3.9×10^-3^	
rs4949397	c	g				0.018			2.4×10^-3^		8.3×10^-3^	3.3×10^-3^			0.010	0.030
rs4949396	t	c				0.020			3.1×10^-3^		8.4×10^-3^	3.9×10^-3^			0.010	0.030
rs1977657	a	t													0.048	
rs4949395	c	t				0.018			4.1×10^-3^		0.011	3.7×10^-3^			0.011	0.030
rs10798849	t	c				0.015			4.5×10^-3^		0.011	3.7×10^-3^			0.015	0.031
rs10753251	g	a	0.019	0.021	0.024										0.012	0.012
rs10798861	g	a	0.015	0.023	0.017										0.012	0.013
rs2839939	c	t		0.035		0.016		0.027	0.010		0.012	4.8×10^-3^	0.035	0.042	2.3×10^-3^	4.4×10^-3^
rs10158864	g	a	0.018	0.022	0.026										0.012	0.015

TH, cortical thickness; SA, cortical surface area; ENG3, ENIGMA3 cohort.

**Table 5B:** p-values for positive associations between disease-risk alleles and TH/SA in brains.

SNP	Risk/Effect allele	SA											TH							
		ENG3	ENG3	ENG3	ENG3	ENG3	ENG3	ENG3	ENG3	ENG3	ENG3	ENG3	UKBB	UKBB	ENG3	ENG3	UKBB	ENG3	ENG3	UKBB
		Bank-ssts	Caudal-top-frontal	insula	Lateral-orbito-frontal	Middle-temporal	Para-central	Pars-oper-cularis	Pars-orbitalis	Pars-tri-angularis	Posterior-cingulate	Pre-central	Caudal-top-frontal	Para-central	Bank-ssts	cuneus	cuneus	Lateral-occipital	Superior-temporal	Superior-parietal
rs12132936	g		0.026						0.033	0.022				0.015	0.017	0.010	0.027			
rs4949403	a		0.021					0.034	0.044	0.028				0.014	9.3×10^-3^	0.010	0.026			0.047
rs4949401	c		0.021					0.026		0.028				0.014	0.010	0.011	0.033			
rs10914383	g		0.024					0.021		0.025				0.020	0.013	0.010	0.027			
rs4949209	c		0.022					0.020	0.035	0.026				0.018	0.012	0.011	0.030			0.047
rs1320584	g		0.024					0.029		0.027				0.016	0.012	0.011	0.029			
rs12037108	c		0.025						0.030	0.024				0.018	0.014	0.010	0.027			
rs10798850	a		0.024											0.019	0.018	8.7×10^-3^	0.031			0.046
rs4478858	t		0.016					0.033	0.027	0.014				0.015	0.016	9.3×10^-3^	0.035			
rs6690908	t	0.049			0.043	0.037						0.028							0.019	
rs12117387	a							5.7×10^-3^	0.030	0.025			0.029	0.035		0.028		0.023		
rs7515829	g							7.3×10^-3^	0.039	0.032			0.037	0.040		0.038		0.024		
rs6425745	g							0.010		0.026			0.034			0.040		0.013		
rs10914374	g							6.9×10^-3^	0.030	0.025			0.027	0.026		0.027		0.028		
rs4949402	t		0.021					0.020	0.035	0.026				0.015	0.010	0.011	0.033			0.049
rs10798848	a							5.6×10^-3^	0.034	0.035			0.035	0.021		0.031		0.026		
rs4949393	g							7.0×10^-3^	0.025	5.3×10^-3^	0.037				0.019					
rs12145450	c							5.4×10^-3^	0.032	0.026			0.029	0.035		0.023		0.023		
rs12563669	a							6.0×10^-3^	0.019	0.010					0.013					
rs7545902	c													0.020			0.031			
rs4949397	c		0.047							9.1×10^-3^				0.049	0.014					
rs4949396	t							0.024	0.024	4.0×10^-3^				0.050	0.014					
rs4949395	c							0.026	0.026	4.2×10^-3^					0.015	0.049				
rs10798849	t							0.022	0.024	5.5×10^-3^					0.015					
rs10753251	g			0.044	0.019		0.019			0.019						0.016		0.019		
rs10798861	g			0.041	0.016		0.018			0.025						0.015		0.019		
rs2839939	c		0.020					0.038	0.012	0.012				0.020	0.025	8.2×10^-3^	0.041			0.044
rs10158864	g			0.036	0.019		0.016			0.020						0.016		0.019		

TH, cortical thickness; SA, cortical surface area; ENG3, ENIGMA3 cohort.

The bipolar-risk alleles decreased TH/SA of precentral and inferior temporal cortices, and increased TH/SA of fusiform, superior parietal, caudal anterior cingulate, rostral anterior cingulate, precuneus, temporal pole, parstriangularis and posterior cingulate cortices (7.8×10^-3^≤p ≤ 0.050; **Table 5C**). Additionally, one autism-risk allele increased TH/SA of fusiform, precuneus, temporal pole, parstriangularis and posteriorcingulate cortices (0.026≤p ≤ 0.047; **Table 5C**).

**Table 5C:** p-values for positive associations between disease-risk alleles and TH/SA in brains.

Associated disorders	SNP	Risk allele	Decreasing TH/SA			Increasing TH/SA by disease-risk alleles								
			Effective allele	ENG3	ENG3	Effective allele	ENG3	ENG3	ENG3	ENG3	ENG3	ENG3	UKBB	UKBB
				SA	TH		SA	SA	TH	TH	SA	TH	TH	TH
				Pre-central	Inferior-temporal		Fusi-form	Superior-parietal	Caudal-anterior-cingulate	Rostral-anterior-cingulate	Pre-cuneus	Temporal-pole	Pars-tri-angularis	Posterior-cingulate
Autism	rs10798856	g	a			g	0.042				0.047	0.033	0.026	0.046
Bipolar	rs12734726	c	t	0.013		c	0.048	0.049	0.012	0.024				
	rs56214663	g	a	0.016	0.046	g		0.021		0.050				
	rs60788028	g	a	0.018	0.036	g		0.017		0.040				
	rs58654289	t	g	0.027	0.041	t		9.1×10^-3^	0.038					
	rs72881860	c	t	0.011		c		0.026		0.049				
	rs4949429	c	g	0.038	0.039	c		9.2×10^-3^	0.038					
	rs4949430	c	t	0.027	0.041	c		9.0×10^-3^	0.038					
	rs59170272	t	c	0.031	0.036	t		0.011	0.036					
	rs72881892	t	a	0.027	0.034	t		8.2×10^-3^	0.037					
	rs10798871	a	g	0.036	0.031	a		7.8×10^-3^	0.029	0.050				

TH, cortical thickness; SA, cortical surface area; ENG3, ENIGMA3 cohort.

## 4 Discussion

As introduced above, we ever phenome-wide scanned a total of 49,268 subjects of European or African descent with 12 different neuropsychiatric disorders and reported that the common *SERINC2* variants and the rare *SERINC2* variant constellations were “specific” to alcoholism in European descent (1, 4). In this study with an expanded sample size of a total of 1,167,439 participants of European, African or Asian descent with 18 diverse neuropsychiatric disorders, and harmonized genetic marker sets, analytical methods, meta-analysis and FDR correction, we confirmed that alcoholism was still the most significant disease associated with *SERINC2* variants in European descent among all neuropsychiatric disorders. Meanwhile, more other substance use disorders that usually are comorbid and share common pathogenesis with alcoholism were also significantly associated with *SERINC2* variants in European descent, including cocaine dependence, marijuana dependence, nicotine dependence, and polysubstance dependence. Additionally, we found that schizophrenia, OCD, and autism in European descent and bipolar disorder in Chinese were also significantly associated with *SERINC2* variants, supporting the findings in literatures. Interestingly, substance use disorders, schizophrenia, OCD and autism but not bipolar disorder had highly similar patterns in association with *SERINC2* variants and regulation by risk *SERINC2* alleles, suggesting potential common mechanism related to *SERINC2* underlying the former four diseases and distinct mechanism from bipolar disorder.

A series of functional studies substantiated the above disease-*SERINC2* associations, which included (i) the significant expression of *SERINC2* mRNA in brain regions, (ii) differential expression of *SERINC2* mRNA in the brains of individuals with alcoholism and schizophrenia compared to controls, and (iii) the regulation of *SERINC2* mRNA expression in the brain, intracranial volume (ICV), subcortical grey matter volumes (GMVs), and cortical surface area (SA) and thickness (TH) by disease-risk alleles. Although much of this nominal functional evidence became only suggestive after correction for multiple testing, a group of suggestive evidence still retains clinical significance. Literature has extensively reported the significant alteration of GMVs in alcoholism, schizophrenia, OCD, autism, and bipolar disorder (24–32). *SERINC2* alleles may play critical roles in the pathogenesis of these diseases via altering the GMVs. Therefore, our conclusion is that *SERINC2* predominantly predisposes individuals to substance dependence, schizophrenia, OCD, autism and bipolar disorder, a conclusion supported not only by statistical evidence but also by biological findings.

Specifically, *SERINC2* mRNA exhibited its highest expression levels in the substantia nigra, followed by the cerebellum (**Figure 1**), and the expression in the substantia nigra and cerebellar hemisphere was down-regulated and up-regulated, respectively, by risk alleles for substance dependence, schizophrenia, OCD, and autism (**Table 3A**). These disease-risk alleles also up-regulated expression in the anterior cingulate, hippocampus, and other cortical regions (**Table 3A**). Additionally, these disease-risk alleles decreased caudate GMV but increased putamen GMV (**Table 4A**). Enlarged putamen GMV has been frequently observed in dopamine-related phenotypes associated with impulsive behaviors, such as substance use disorders (24), schizophrenia (25, 26), autism (27, 28), and OCD (28–31). This supports our hypothesis that *SERINC2* alleles may increase the risk for these disorders by enlarging putamen GMV.

In contrast, bipolar disorder-risk alleles down-regulated *SERINC2* mRNA expression in the caudate, hypothalamus, and cerebellar hemisphere, while up-regulating it in the frontal cortex (**Table 3B**). These bipolar-risk alleles also decreased caudate and pallidum GMVs (**Table 4B**), consistent with reports of reduced GMVs in these regions in bipolar disorder (33–36). This supports the hypothesis that *SERINC2* alleles may increase the risk for bipolar disorder by reducing caudate and pallidum GMVs. Additionally, one autism-risk allele down-regulated mRNA expression in both the cerebellar hemisphere and cortex (**Table 3B**).

An alcoholism-risk allele was also found to decrease ICV (**Table 4A**), aligning with evidence of widespread brain shrinkage in alcoholism (37–41), supporting the hypothesis that this *SERINC2* allele may increase the risk for alcoholism by reducing brain volume. Furthermore, several schizophrenia-risk alleles were associated with increased accumbens GMVs (**Table 4A**), consistent with prior findings of enlarged nucleus accumbens GMV in schizophrenia (42). This suggests that *SERINC2* alleles may contribute to schizophrenia risk through accumbens GMV enlargement. Lastly, the disease-risk *SERINC2* alleles were found to regulate the SA/TH of various cortical regions (**Table 5**), consistent with previous reports of cortical alterations in psychiatric disorders, such as schizophrenia (43, 44). This supports the idea that *SERINC2* alleles may play key roles in the pathogenesis of these psychiatric diseases by altering cortical SA/TH too.

The sharing of risk *SERINC2* variants and their functional patterns among alcoholism, cocaine dependence, marijuana dependence, nicotine dependence, polysubstance dependence, schizophrenia, OCD, and autism may be interpreted by the high comorbidity rates among these diseases. For example, there is a higher incidence of alcoholism in the family members of ASD patients compared with the general population; also, there is a link between the autism susceptibility candidate 2 gene (*AUTS2*) in the regulation of alcohol consumption (45–47).

In summary, these findings indicate that *SERINC2* is primarily linked to substance dependence, schizophrenia, OCD, autism and bipolar disorder, a conclusion that is supported by both statistical and biological evidence and published literatures.

## Data Availability

The datasets used for the analyses described in this manuscript were obtained from dbGaP. The dbGaP accession numbers, PIs’ names, grant numbers and references are listed in **Supplementary Table S1**.

## References

[1] Zuo LJ Wang KS Zhang XY Krystal JH Li CSR Zhang FY. NKAIN1-SERINC2 is a functional, replicable and genome-wide significant risk gene region specific for alcohol dependence in subjects of European descent. Drug Alcohol Depend. (2013) 129:254–64. doi: 10.1016/j.drugalcdep.2013.02.006 PMC3628730 23455491

[2] Zuo L Tan Y Zhang X Wang X Krystal J Tabakoff B. A new genomewide association meta-analysis of alcohol dependence. Alcohol Clin Exp Res. (2015) 39:1388–95. doi: 10.1111/acer.12786 PMC5587504 26173551

[3] Zuo L Lu L Tan Y Pan X Cai Y Wang X. Genome-wide association discoveries of alcohol dependence. Am J Addict. (2014) 23:526–39. doi: 10.1111/j.1521-0391.2014.12147.x PMC4187224 25278008

[4] Zuo L Wang KS Zhang XY Li CS Zhang F Wang X. Rare SERINC2 variants are specific for alcohol dependence in individuals of European descent. Pharmacogenet Genomics. (2013) 23:395–402. doi: 10.1097/FPC.0b013e328362f9f2 23778322 PMC4287355

[5] Yang D Chen J Cheng X Cao B Chang H Li X. SERINC2 increases the risk of bipolar disorder in the Chinese population. Depress Anxiety. (2021) 38:985–95. doi: 10.1002/da.23186 34288243

[6] Hnoonual A Thammachote W Tim-Aroon T Rojnueangnit K Hansakunachai T Sombuntham T. Chromosomal microarray analysis in a cohort of underrepresented population identifies SERINC2 as a novel candidate gene for autism spectrum disorder. Sci Rep. (2017) 7:12096. doi: 10.1038/s41598-017-12317-3 28935972 PMC5608768

[7] Inuzuka M Hayakawa M Ingi T. Serinc, an activity-regulated protein family, incorporates serine into membrane lipid synthesis. J Biol Chem. (2005) 280:35776–83. doi: 10.1074/jbc.M505712200 16120614

[8] Guirland C Suzuki S Kojima M Lu B Zheng JQ. Lipid rafts mediate chemotropic guidance of nerve growth cones. Neuron. (2004) 42:51–62. doi: 10.1016/S0896-6273(04)00157-6 15066264

[9] Howie BN Donnelly P Marchini J. A flexible and accurate genotype imputation method for the next generation of genome-wide association studies. PloS Genet. (2009) 5:e1000529. doi: 10.1371/journal.pgen.1000529 19543373 PMC2689936

[10] Zuo L Wang K Zhang XY Pan X Wang G Tan Y. Association between common alcohol dehydrogenase gene (ADH) variants and schizophrenia and autism. Hum Genet. (2013) 132:735–43. doi: 10.1007/s00439-013-1277-4 PMC3683370 23468174

[11] Zuo L Gelernter J Zhang CK Zhao H Lu L Kranzler HR. Genome-wide association study of alcohol dependence implicates KIAA0040 on chromosome 1q. Neuropsychopharmacology. (2012) 37:557–66. doi: 10.1038/npp.2011.229 PMC3242317 21956439

[12] Purcell S Neale B Todd-Brown K Thomas L Ferreira MA Bender D. PLINK: a tool set for whole-genome association and population-based linkage analyses. Am J Hum Genet. (2007) 81:559–75. doi: 10.1086/519795 PMC1950838 17701901

[13] Storey J Bass A Dabney A Robinson D. qvalue: Q-value estimation for false discovery rate control. *R package version 2.32.0.* (2023). Available online at: http://github.com/jdstorey/qvalue (Accessed December 9, 2024).

[14] GTEx Consortium. The genotype-tissue expression (GTEx) project. Nat Genet. (2013) 45:580–5. doi: 10.1038/ng.2653 PMC4010069 23715323

[15] McClintick JN Xuei X Tischfield JA Goate A Foroud T Wetherill L. Stress-response pathways are altered in the hippocampus of chronic alcoholics. Alcohol. (2013) 47:505–15. doi: 10.1016/j.alcohol.2013.07.002 PMC3836826 23981442

[16] Bannon MJ Johnson MM Michelhaugh SK Hartley ZJ Halter SD David JA. A molecular profile of cocaine abuse includes the differential expression of genes that regulate transcription, chromatin, and dopamine cell phenotype. Neuropsychopharmacology. (2014) 39:2191–9. doi: 10.1038/npp.2014.70 PMC4104338 24642598

[17] Philibert RA Ryu GY Yoon JG Sandhu H Hollenbeck N Gunter T. Transcriptional profiling of subjects from the Iowa adoption studies. Am J Med Genet B Neuropsychiatr Genet. (2007) 144B:683–90. doi: 10.1002/ajmg.b.30512 17342724

[18] Ryan MM Lockstone HE Huffaker SJ Wayland MT Webster MJ Bahn S. Gene expression analysis of bipolar disorder reveals downregulation of the ubiquitin cycle and alterations in synaptic genes. Mol Psychiatry. (2006) 11:965–78. doi: 10.1038/sj.mp.4001875 16894394

[19] Brennand KJ Simone A Jou J Gelboin-Burkhart C Tran N Sangar S. Modelling schizophrenia using human induced pluripotent stem cells. Nature. (2011) 473:221–5. doi: 10.1038/nature09915 PMC3392969 21490598

[20] Barnes MR Huxley-Jones J Maycox PR Lennon M Thornber A Kelly F. Transcription and pathway analysis of the superior temporal cortex and anterior prefrontal cortex in schizophrenia. J Neurosci Res. (2011) 89:1218–27. doi: 10.1002/jnr.22647 21538462

[21] Maycox PR Kelly F Taylor A Bates S Reid J Logendra R. Analysis of gene expression in two large schizophrenia cohorts identifies multiple changes associated with nerve terminal function. Mol Psychiatry. (2009) 14:1083–94. doi: 10.1038/mp.2009.18 19255580

[22] Harris LW Wayland M Lan M Ryan M Giger T Lockstone H. The cerebral microvasculature in schizophrenia: a laser capture microdissection study. PloS One. (2008) 3:e3964. doi: 10.1371/journal.pone.0003964 19088852 PMC2597747

[23] Guo X Luo X Zhang Y Yu Z Tan Z Cao L. Phenome-wide association studies of TXNRD2-COMT-ARVCF cluster pinpoint schizophrenia and bipolar disorder. Asian J Psychiatr. (2024) 98:104145. doi: 10.1016/j.ajp.2024.104145 38959548

[24] Ersche KD Jones PS Williams GB Turton AJ Robbins TW Bullmore ET. Abnormal brain structure implicated in stimulant drug addiction. Science. (2012) 335:601–4. doi: 10.1126/science.1214463 22301321

[25] Buchsbaum MS Shihabuddin L Brickman AM Miozzo R Prikryl R Shaw R. Caudate and putamen volumes in good and poor outcome patients with schizophrenia. Schizophr Res. (2003) 64:53–62. doi: 10.1016/S0920-9964(02)00526-1 14511801

[26] Hokama H Shenton ME Nestor PG Kikinis R Levitt JJ Metcalf D. Caudate, putamen, and globus pallidus volume in schizophrenia: a quantitative MRI study. Psychiatry Res. (1995) 61:209–29. doi: 10.1016/0925-4927(95)02729-H 8748466

[27] Sato W Kubota Y Kochiyama T Uono S Yoshimura S Sawada R. Increased putamen volume in adults with autism spectrum disorder. Front Hum Neurosci. (2014) 8:957. doi: 10.3389/fnhum.2014.00957 25505401 PMC4243557

[28] Hollander E Anagnostou E Chaplin W Esposito K Haznedar MM Licalzi E. Striatal volume on magnetic resonance imaging and repetitive behaviors in autism. Biol Psychiatry. (2005) 58:226–32. doi: 10.1016/j.biopsych.2005.03.040 15939406

[29] Radua J van den Heuvel OA Surguladze S Mataix-Cols D. Meta-analytical comparison of voxel-based morphometry studies in obsessive-compulsive disorder vs other anxiety disorders. Arch Gen Psychiatry. (2010) 67:701–11. doi: 10.1001/archgenpsychiatry.2010.70 20603451

[30] Radua J Mataix-Cols D. Voxel-wise meta-analysis of grey matter changes in obsessive-compulsive disorder. Br J Psychiatry. (2009) 195:393–402. doi: 10.1192/bjp.bp.108.055046 19880927

[31] Hibar DP Cheung JW Medland SE Mufford MS Jahanshad N Dalvie S. Significant concordance of genetic variation that increases both the risk for obsessive-compulsive disorder and the volumes of the nucleus accumbens and putamen. Br J Psychiatry. (2018) 213:430–6. doi: 10.1192/bjp.2018.62 PMC6053271 29947313

[32] Luo X Mao Q Shi J Wang X Li CR. Putamen gray matter volumes in neuropsychiatric and neurodegenerative disorders. World J Psychiatry Ment Health Res. (2019) 3:pii:1020. PMC6641567 31328186

[33] Beyer JL Kuchibhatla M Payne M Moo-Young M Cassidy F MacFall J. Caudate volume measurement in older adults with bipolar disorder. Int J Geriatr Psychiatry. (2004) 19:109–14. doi: 10.1002/gps.1030 14758576

[34] Janiri D Sani G Rossi P Piras F Iorio M Banaj N. Amygdala and hippocampus volumes are differently affected by childhood trauma in patients with bipolar disorders and healthy controls. Bipolar Disord. (2017) 19:353–62. doi: 10.1111/bdi.12516 28699182

[35] McWhinney SR Abe C Alda M Benedetti F Boen E Del Mar Bonnin C. Association between body mass index and subcortical brain volumes in bipolar disorders-ENIGMA study in 2735 individuals. Mol Psychiatry. (2021) 26:6806–19. doi: 10.1038/s41380-021-01098-x PMC8760047 33863996

[36] Zhang X Gao W Cao W Kuang L Niu J Guo Y. Pallidal volume reduction and prefrontal-striatal-thalamic functional connectivity disruption in pediatric bipolar disorders. J Affect Disord. (2022) 301:281–8. doi: 10.1016/j.jad.2022.01.049 35031334

[37] Chandrasekar R. Alcohol and NMDA receptor: current research and future direction. Front Mol Neurosci. (2013) 6:14. doi: 10.3389/fnmol.2013.00014 23754976 PMC3664776

[38] Dager AD McKay DR Kent JW Jr. Curran JE Knowles E Sprooten E. Shared genetic factors influence amygdala volumes and risk for alcoholism. Neuropsychopharmacology. (2015) 40:412–20. doi: 10.1038/npp.2014.187 PMC4443955 25079289

[39] Le Berre AP Rauchs G La Joie R Mezenge F Boudehent C Vabret F. Impaired decision-making and brain shrinkage in alcoholism. Eur Psychiatry. (2014) 29:125–33. doi: 10.1016/j.eurpsy.2012.10.002 23182846

[40] Tongsong T Puntachai P Mekjarasnapha M Traisrisilp K. Severe fetal brain shrinkage following heavy maternal alcohol consumption. Ultrasound Obstet Gynecol. (2014) 44:245–7. doi: 10.1002/uog.13396 24777961

[41] Zhang K Luo J. Role of MCP-1 and CCR2 in alcohol neurotoxicity. Pharmacol Res. (2019) 139:360–6. doi: 10.1016/j.phrs.2018.11.030 PMC6360095 30472461

[42] Lauer M Senitz D Beckmann H. Increased volume of the nucleus accumbens in schizophrenia. J Neural Transm (Vienna). (2001) 108:645–60. doi: 10.1007/s007020170042 11478417

[43] Mao Q Lin X Yin Q Liu P Zhang Y Qu S. A significant, functional and replicable risk KTN1 variant block for schizophrenia. Sci Rep. (2023) 13:3890. doi: 10.1038/s41598-023-27448-z 36890161 PMC9995530

[44] Guo X Luo X Huang X Zhang Y Ji J Wang X. The role of 3' Regulatory region flanking kinectin 1 gene in schizophrenia. Alpha Psychiatry. (2024) 25:413–20. doi: 10.5152/alphapsychiatry.2024.241616 PMC11322729 39148597

[45] Miles JH Takahashi TN Haber A Hadden L. Autism families with a high incidence of alcoholism. J Autism Dev Disord. (2003) 33:403–15. doi: 10.1023/a:1025010828304 12959419

[46] Regier DA Farmer ME Rae DS Locke BZ Keith SJ Judd LL. Comorbidity of mental disorders with alcohol and other drug abuse. Results from the Epidemiologic Catchment Area (ECA) Study. Jama. (1990) 264:2511–8. doi: 10.1001/jama.1990.03450190043026 2232018

[47] Klimkiewicz A Klimkiewicz J Jakubczyk A Kieres-Salomonski I Wojnar M. Comorbidity of alcohol dependence with other psychiatric disorders. Part I. Epidemiol dual diagnosis]. Psychiatr Pol. (2015) 49:265–75. doi: 10.12740/PP/25704 26093591

